# Cognitive decline due to excess synaptic Zn^2+^ signaling in the hippocampus

**DOI:** 10.3389/fnagi.2014.00026

**Published:** 2014-02-27

**Authors:** Atsushi Takeda, Haruna Tamano

**Affiliations:** Department of Bioorganic Chemistry, School of Pharmaceutical Sciences, University of ShizuokaShizuoka, Japan

**Keywords:** Zn^2^^+^ signal, hippocampus, cognition, glucocorticoid, glutamate

## Abstract

Zinc is an essential component of physiological brain function. Vesicular zinc is released from glutamatergic (zincergic) neuron terminals and serves as a signal factor (Zn^2^^+^ signal) in both the intracellular (cytosol) compartment and the extracellular compartment. Synaptic Zn^2^^+^ signaling is dynamically linked to neurotransmission and is involved in processes of synaptic plasticity such as long-term potentiation and cognitive activity. On the other hand, the activity of the hypothalamic–pituitary–adrenal (HPA) axis, i.e., glucocorticoid secretion, which can potentiate glutamatergic neuron activity, is linked to cognitive function. HPA axis activity modifies synaptic Zn^2^^+^ dynamics at zincergic synapses. An increase in HPA axis activity, which occurs after exposure to stress, may induce excess intracellular Zn^2^^+^ signaling in the hippocampus, followed by hippocampus-dependent memory deficit. Excessive excitation of zincergic neurons in the hippocampus can contribute to cognitive decline under stressful and/or pathological conditions. This paper provides an overview of the ``Hypothesis and Theory'' of Zn^2^^+^-mediated modification of cognitive activity.

## INTRODUCTION

Over 300 proteins require zinc to carry out their functions in microorganisms, plants, and animals. Zinc powerfully influences cell division and differentiation (Vallee and Falchuk, 1993; Maret and Sandstead, 2008; Prasad, 2008). Zinc is also essential for the growth and functioning of the brain. Zinc transport from the plasma to the brain’s extracellular fluid and cerebrospinal fluid is strictly regulated by the brain-barrier system, i.e., the blood–brain and blood-CSF barrier. The brain barrier system maintains zinc homeostasis in the brain (Takeda, 2000, 2001). Zinc homeostasis is critical for brain function (Capasso et al., 2005; Mocchegiani et al., 2005) and is spatiotemporally altered in the process of neurological diseases (Barnham and Bush, 2008).

**FIGURE 1:**
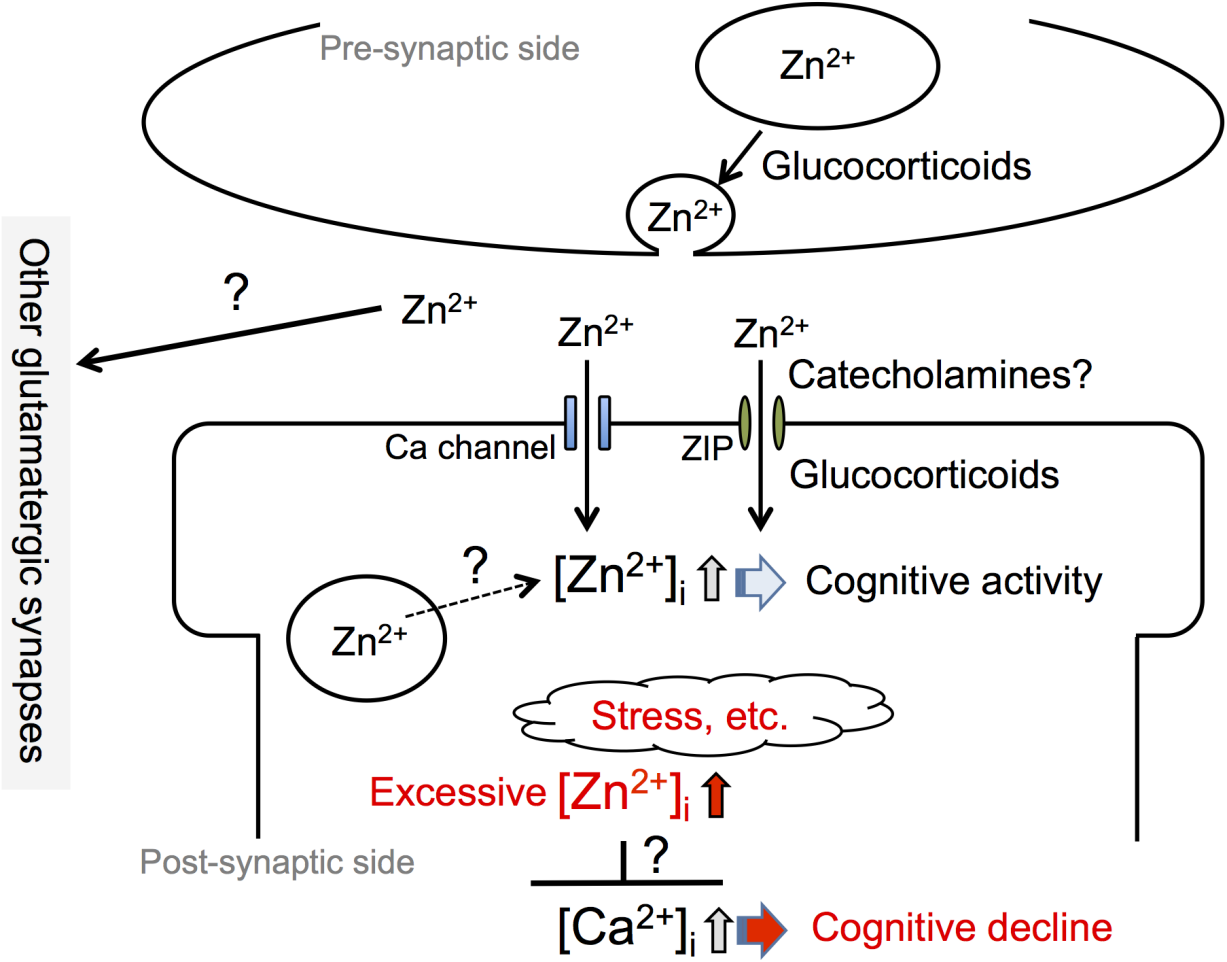
**Involvement of synaptic Zn^2+^ dynamics in cognitive activity**. An increase in intracellular Zn^2^^+^ concentration, [Zn^2^^+^]_i_, which is induced by an influx of extracellular Zn^2^^+^ at zincergic synapses in the hippocampus, is involved in cognitive activity. Presynaptic glucocorticoid signaling, a non-genomic action, and post-synaptic glucocorticoid signaling, a genomic action, modify the degree of increase in intracellular Zn^2^^+^. It is also possible that catecholamines modify the degree through the activity of the β-adrenergic system. The degree of increase in intracellular Zn^2^^+^ is linked to cognitive activity and excess intracellular Zn^2^^+^ signaling, which can be induced by stress, is involved in cognitive decline. The excess might affect intracellular Ca^2^^+^ signaling, which plays a key role for synaptic plasticity.

Zinc is relatively concentrated in the hippocampus and amygdala (Takeda et al., 1995). Both regions are enriched with histochemically reactive zinc, as revealed by Timm’s sulfide-silver staining method (Frederickson, 1989; Frederickson and Danscher, 1990). Histochemically reactive zinc is found predominantly in the presynaptic vesicles and serves as a signal factor (Zn^2^^+^ signal) in both the cytosolic and extracellular compartments. Zn^2^^+^ is released with glutamate in a calcium-dependent and impulse-dependent manner from glutamatergic (zincergic) neuron terminals (**Figure 1**). Zn^2^^+^ released from these terminals modulates the activity of several important receptors, including the α-amino-3-hydroxy-5-methyl-4-isoxazolepropionic acid (AMPA)/kainate receptor, *N*-methyl-D-aspartate (NMDA) receptors, and γamino butyric acid (GABA) receptors in the extracellular compartment (Smart et al., 1994; Nakashima and Dyck, 2009), and is taken up into post-synaptic neurons to serve as an intracellular signal factor. Glutamatergic (zincergic) circuits play a key role in cognitive map building structures such as the hippocampus (Martinez-Guijarro et al., 1991; Nacher et al., 2000). It has been estimated that approximately 20% of total brain zinc is histochemically reactive, based on the finding that the removal of zinc transporter-3 (ZnT3) protein, which is responsible for the movement of zinc from the cytoplasm into synaptic vesicles (Palmiter et al., 1996), results in a 20% reduction of the total amount of zinc in the brain (Cole et al., 1999).

It is well known that the hippocampus and amygdala are involved in cognitive and emotional behavior. Synaptic plasticity such as long-term potentiation (LTP) is believed to be a key cellular mechanism involved in learning and memory and has been widely studied in relation to glutamatergic synapses in the brain, especially in the hippocampus (Bliss and Collingridge, 2013). When information is processed in memory, glutamatergic neurons form a neural circuit in the hippocampus and the amygdala. Furthermore, it has been reported that plastic changes in hippocampal synapses occur activity-dependently during the performance of associative learning tasks (Gruart et al., 2006; Clarke et al., 2010).

On the other hand, the activity of the hypothalamic–pituitary–adrenal (HPA) axis, i.e., glucocorticoid secretion, is linked to cognitive and emotional functions and can potentiate glutamatergic neuron activity (Sandi, 2011). There is some evidence that the modification of synaptic Zn^2^^+^ signaling by HPA axis activity, which is enhanced by stress and aging, is linked to cognitive and emotional behavior, and that abnormal modification may induce cognitive decline (Takeda and Tamano, 2009, 2010, 2012). It is well known that abnormal Zn^2^^+^ influx into post-synaptic neurons, which is induced by abnormal glutamatergic (zincergic) neuron activity, induces neuronal death and is involved in neurological disorders such as stroke/ischemia and temporal lobe epilepsy (Frederickson et al., 2005; Sensi et al., 2011; Takeda, 2011a; Weiss, 2011). Therefore, the homeostasis of synaptic Zn^2^^+^ signaling is critical in both functional and pathological aspects (Takeda, 2011b; Takeda et al., 2013). On the basis of recent evidence that excessive excitation of zincergic neurons in the hippocampus can contribute to cognitive decline under stressful and/or pathological conditions (Takeda et al., 2009, 2011, 2012), this paper provides an overview of the “Hypothesis and Theory” of Zn^2^^+^-mediated modification of cognitive activity.

## SYNAPTIC Zn^2+^ HOMEOSTASIS

Total zinc concentration in the adult brain reaches around 200 μM (Markesbery et al., 1984). Extracellular zinc concentration in the adult brain is estimated to be less than 1 μM (Weiss et al., 2000). If zinc concentration in the brain’s extracellular fluid is equal to that in cerebrospinal fluid (Hershey et al., 1983), it is around 150 nM – approximately one thousandth of total brain zinc concentration. In zincergic synapses, Zn^2^^+^ concentration in the synaptic cleft is estimated to be higher than that in the brain’s (extrasynaptic) extracellular fluid, because under hippocampal-slice-experiment conditions the regions where zincergic synapses are found are intensely stained by ZnAF-2, a membrane-impermeable zinc indicator (Minami et al., 2006). The synaptic cleft is surrounded with the processes of astrocytes, which contribute to maintaining a steady concentration of zinc and neurotransmitters in the cleft. Interestingly, Zn^2^^+^ level in the brain’s extracellular fluid, which is estimated to be approximately 20 nM (Frederickson et al., 2006), is higher than that in the plasma (<1 nM; Magneson et al., 1987). In the brain’s extracellular fluid, the high ratio of Zn^2^^+^ concentration to total zinc concentration appears to be associated with the synaptic Zn^2^^+^ dynamics of the brain. There is some evidence that extracellular Zn^2^^+^ serves as a pool for the zinc in the synaptic vesicle and is involved in synaptic Zn^2^^+^ homeostasis (Takeda et al., 2006), although the chemical form of this vesicular zinc is unknown.

Basal Zn^2^^+^ concentration is extremely low in the intracellular (cytosol) compartment (<1 nM; Sensi et al., 1997; Colvin et al., 2008). ZnT proteins such as ZnT1, ZnT3, and ZnT10, and Zrt-Irt-like proteins (ZIP) such as ZIP4 and ZIP6 are involved in the control of Zn^2^^+^ levels in the cytosolic compartment, especially under static (basal) conditions (Emmetsberger et al., 2010). Some of these transporters transport cytosolic Zn^2^^+^ into a variety of subcellular organelles, including mitochondria, lysosomes, endosomes, and the Golgi apparatus, probably to maintain static Zn^2^^+^ levels in the cytosolic compartment (Sensi et al., 2003; Danscher and Stoltenberg, 2005; Colvin et al., 2006). On the other hand, it is possible that Zn^2^^+^ release from subcellular organelles, which might be induced by synaptic glutamate signaling, is involved in Zn^2^^+^ signaling (Stork and Li, 2010). Zn^2^^+^ levels other than vesicular zinc serving as Zn^2^^+^ are estimated to be less than 5% of the total amount of Zn^2^^+^ in the hippocampus and cerebral cortex (Lee et al., 2011). ZnT1 is a major Zn^2^^+^ transporter in the plasma membrane and may be involved in cytosolic Zn^2^^+^ homeostasis in neurons by transporting Zn^2^^+^ from the somata to the extracellular space (Sekler et al., 2002). It has been reported that ZnT1 prevents excessive accumulation of Zn^2^^+^ in the cytosolic compartment (Nolte et al., 2004), resulting in the protection of neurons from Zn^2^^+^ toxicity in neurological diseases such as transient forebrain ischemia (Aguilar-Alonso et al., 2008). Tissue plasminogen activator, a secreted serine protease, is excitotoxic and increases lysosomal sequestration of increased Zn^2^^+^ in the cytosolic compartment through interaction with ZIP4, which may also contribute to the protection of neurons from Zn^2^^+^ toxicity (Emmetsberger et al., 2010). The spatiotemporal control of Zn^2^^+^ signaling via ZIP and ZnT maintains a steady-state environment in both the extracellular and cytosolic compartments (Fukada and Kambe, 2011).

## FUNCTIONAL AND NEUROTOXIC Zn^2+^ SIGNALING

Zn^2^^+^ concentration is increased in the synaptic cleft during the excitation of zincergic synapses, followed by an increase in the cytosol (intracellular compartment; **Figure 1**). Released Zn^2^^+^ is quickly taken up into presynaptic and post-synaptic neurons and astrocytes. Calcium channels such as calcium-permeable AMPA/kainate receptors are involved in Zn^2^^+^ influx during neuronal excitation (Weiss et al., 2000; Jia et al., 2002; Takeda et al., 2007a). The increase in the extracellular concentration of Zn^2^^+^ is dependent on the frequency of depolarizing stimulation (Ueno et al., 2002). Therefore, the increase in intracellular concentration of Zn^2^^+^ serving as a signal factor is closely correlated to zincergic neuron excitation (Takeda et al., 2013).

Glutamate accumulates in the extracellular compartment due to excessive excitation of glutamatergic (zincergic) neurons. Excessive activation of glutamate receptors caused by excess extracellular glutamate leads to a number of deleterious consequences, including impairment of calcium buffering, generation of free radicals, activation of mitochondrial permeability transition, and secondary excitotoxicity (Danbolt, 2001; Dong et al., 2009). Glutamate excitotoxicity, a final common pathway for neuronal death, is observed in numerous pathological processes such as stroke/ischemia, temporal lobe epilepsy, Alzheimer’s disease, and amyotrophic lateral sclerosis. An excess of extracellular Zn^2^^+^, which is induced under glutamate excototoxicity, is harmful; excessive Zn^2^^+^ influx into post-synaptic neurons is involved in neurodegeneration under pathological conditions. Calcium-permeable AMPA receptors may play a key role in this Zn^2^^+^ influx (Liu et al., 2004; Noh et al., 2005; Weiss, 2011).

Zn^2^^+^ also plays a neuroprotective role in glutamate-induced excitotoxicity by activating pre-synaptic ATP-sensitive potassium channels and by inhibiting GABA transporter 4 (Bancila et al., 2004; Cohen-Kfir et al., 2005). It is estimated that the neuroprotective action of Zn^2^^+^ occurs under conditions in which zincergic neurons are not excessively excited. Zn^2^^+^ released from zincergic neuron terminals may also serve as a negative feedback factor against glutamate release (Minami et al., 2006; Takeda et al., 2007b). Therefore, the degree of increase in extracellular Zn^2^^+^ is critical in both functional and neurotoxic aspects.

## Zn^2+^ SIGNALING AND COGNITION

Synaptic Zn^2^^+^ signaling is involved in processes of synaptic plasticity such as LTP in the hippocampus and amygdala. Enhanced plasticity in zincergic synapses is associated with cortical modification after exposure to an enriched environment (Nakashima and Dyck, 2008). The enhanced plasticity of zincergic synapses in the hippocampus underlies the acquisition of new motor and cognitive abilities (Delgado-García and Gruart, 2006; Jurado-Parras et al., 2013). These findings suggest that synaptic Zn^2^^+^ signaling is involved in cognitive and emotional behavior through the modulation of synaptic plasticity such as LTP (**Figure 1**).

Targeted deletion of the ZnT3 prevents vesicular Zn^2^^+^ uptake (Cole et al., 1999) and ablates Zn^2^^+^ release into the extracellular space by action potentials. There is a correlation between vesicular Zn^2^^+^ levels and ZnT3 protein expression (Palmiter et al., 1996). Zn^2^^+^ transport into the synaptic vesicle is ZnT3-dependent and is important for amassing the large pool of Zn^2^^+^ used in signaling (Lee et al., 2011). It has been reported that Zn^2^^+^ signaling is involved in cognitive and emotional behavior even in ZnT3KO (Adlard et al., 2010; Martel et al., 2010, 2011; Sindreu et al., 2011). The pool of Zn^2^^+^ may be located in other subcellular organelles (**Figure 1**) and/or zinc-binding proteins such as metallothionein in ZnT3KO mice. On the other hand, memory deficit and the changes in emotional (freezing) behavior have been observed in wild-type animals when acute loss or chelation of synaptic Zn^2^^+^ is induced by treatment with zinc chelators (Takeda et al., 2010a, b). The amount of Zn^2^^+^ functioning as a signal factor seems to be lower in ZnT3KO mice than in wild-type mice.

Saito et al. (2000) report that age-dependent reduction of Zn^2^^+^ levels in the synaptic vesicles of the mossy fibers induced by low ZnT3 expression causes both glutamatergic excitotoxicity in hippocampal neurons and the deterioration of learning and memory in senescence-accelerated mouse prone 10 (SAMP10). There are also reports of age-dependent reductions in ZnT3 expression and synaptic Zn^2^^+^ levels in the hippocampal mossy fibers of human amyloid precursor protein-transgenic (Tg2576) mice, suggesting that extensive modifications of the brain’s Zn^2^^+^ pool, particularly synaptic (vesicular) Zn^2^^+^, underlie the neuronal dysfunction characteristic of Alzheimer’s disease (Lee et al., 2012). Furthermore, there is a significant age-related decline in cortical ZnT3 levels from age 48 to 91 in healthy people (Adlard et al., 2010) and ZnT3 levels are more markedly decreased in the cortex in Alzheimer’s disease. It is likely that the increase in extracellular Zn^2^^+^ induced by the physiological excitation of zincergic neurons requires cognitive activity (**Figure 1**) and that an insufficient increase is involved in the pathophysiology of Alzheimer’s disease.

On the other hand, Zn^2^^+^ released from zincergic neurons is known to mediate parenchymal and cerebrovascular amyloid formation in Tg2576 mice (Lee et al., 2002; Friedlich et al., 2004; Stoltenberg et al., 2007). The transsynaptic movement of Zn^2^^+^ may be severely compromised in Alzheimer’s disease, both by lack of ZnT3 expression and by sequestration in amyloid. Adlard et al. (2010) report that the genetic ablation of ZnT3 may represent a phenocopy for memory deficits in Alzheimer’s disease. Deshpande et al. (2009) postulate that the sequestration of Zn^2^^+^ in oligomeric amyloid-β (Aβ)–Zn complexes may lead to a reduction in Zn^2^^+^ availability at the synapses, resulting in a loss of the modulatory activity of Zn^2^^+^, and leading to the cognitive decline of Alzheimer’s disease. Such changes in synaptic Zn^2^^+^ availability may participate in modifying cognitive activity and also in cognitive decline (**Figure 1**; Linkous et al., 2009; Bush, 2013; Bosomworth et al., 2013).

## GLUCOCORTICOID SIGNALING, Zn^2+^ SIGNALING, AND COGNITION

The hippocampus is enriched with corticosteroid receptors and is the major target region of corticosteroids (Joëls, 2008). Mineralocorticoid receptors and glucocorticoid receptors are colocalized in CA1 and CA2 pyramidal cells and in dentate gyrus granule cells. In CA3 pyramidal cells, on the other hand, mineralocorticoid receptors are abundantly expressed, while glucocorticoid receptors are expressed at much lower levels (Ozawa, 2005). Mineralocorticoid receptors are extensively occupied with low levels of corticosterone, and glucocorticoid receptors are particularly activated after exposure to stress (Joëls, 2008; Sandi, 2011).

An increase in serum corticosterone level induces a rapid increase in hippocampal corticosterone level, in parallel with an increase in extracellular glutamate level (Venero and Borrell, 1999). Corticosterone-induced increase in extracellular glutamate levels in the hippocampus appears to be exerted through the action of membrane-associated mineralocorticoid receptors and/or glucocorticoid receptors, which increase the probability of glutamate release in synaptic activation (Karst et al., 2005; Musazzi et al., 2010). The rapid effects of corticosterone on glutamatergic transmission appear to be linked to diverse effects on synaptic plasticity and memory processes in the hippocampus (**Figure 1**). An increase in the probability of glutamate release through the action of corticosterone leads to increases both in the amount of glutamate released during learning and in the degree of activation of post-synaptic glutamate receptors. Corticosterone can contribute to an increase in the efficacy of glutamatergic transmission by AMPA receptor insertion at synaptic sites, through both the rapid and the delayed (genomic) effects. These effects are of advantage to processes of synaptic plasticity such as LTP and memory acquisition (Sandi, 2011). Therefore, it is estimated that corticosterone increases the probability of Zn^2^^+^ release from zincergic neuron terminals through the rapid non-genomic effect in the hippocampus (**Figure 1**; Takeda et al., 2012). Futhermore, corticosterone requires intracellular Zn^2^^+^ signaling for the genomic effect, possibly followed by the delayed influx of extracellular Zn^2^^+^ through zinc transport systems such as ZIP (**Figure 1**). Although the evidence is limited, it is likely that synaptic Zn^2^^+^ signaling cooperates with corticosteroid signaling in learning and memory.

In contrast, glutamate accumulates in the extracellular compartment at high levels through a corticosterone-mediated blockade of glutamate transporter activity when corticosterone is abnormally secreted under conditions of severe stress. Abnormal corticosterone secretion also contributes to abnormal glutamate release from neuron terminals (Wong et al., 2007; Howland and Wang, 2008). The extracellular spillover of glutamate impairs spatial memory retrieval. Furthermore, Wong et al. (2007) demonstrate that hippocampal long-term depression (LTD) is both necessary and sufficient to cause acute stress-induced impairment of spatial memory retrieval. Excess intracellular Zn^2^^+^ signaling induced by corticosterone and/or stress is also involved in the impairment of hippocampal LTP (Takeda et al., 2009, 2012), possibly followed by the impairment of learning and memory (Takeda et al., 2011; **Figure 1**). In hippocampal CA3, on the other hand, an increase in intracellular Zn^2^^+^ via a zinc ionophore not only decreases basal Ca^2^^+^ level but also suppresses increases in Ca^2^^+^ level via metabotropic glutamate receptors (Takeda et al., 2007a). Such excess intracellular Zn^2^^+^ signaling may lead to negative crosstalk in intracellular Ca^2^^+^ signaling, which plays a key role in LTP and LTD (**Figure 1**).

A selective increase in the nocturnal levels of corticol has been observed in aged humans (Landfield and Eldridge, 1994). Furthermore, high levels of cortisol are found in Alzheimer’s disease as well as in depression. In Alzheimer’s disease patients, cognitive deficits (such as in memory) and psychological symptoms (such as anxiety) are associated with an early deregulation of the HPA axis (Swanwick et al., 1998; Brureau et al., 2013). Therefore, it is possible that excess intracellular Zn^2^^+^ signaling through abnormal cortisol secretion is involved in cognitive deficits in both normal aging and neurological diseases such as dementia.

On the other hand, corticotrophin releasing hormone (CRH) drives the HPA axis and is considered to be the central coordinator of behavioral, autonomic, and neuroendocrine stress responses. The stress mediators activated by CRH are organized in the sympathetic nervous system, as well as in the HPA axis (de Kloet, 2008). Adrenaline, along with norepinephrine, is largely responsible for the immediate reactions that are felt under conditions of stress. Responses of adrenaline and norepinephrine, such as an increase in heart rate, occur more quickly than those of glucocorticoids. Catecholamines are released from the sympathetic nerve system and the adrenal glad. It has been reported that the enhanced memory associated with emotional experiences involves activation of the β-adrenergic system (Cahill et al., 1994; McEwen and Sapolsky, 1995). β-Adrenergic receptor activation facilitates the induction of a protein synthesis-dependent late phase in LTP in the hippocampus (Gelinas and Nguyen, 2005). Learning-facilitated LTD and LTP at mossy fiber-CA3 synapses requires activation of β-adrenergic receptors (Hagena and Manahan-Vaughan, 2012). The above evidence suggests that synaptic Zn^2^^+^ signaling is modified by the β-adrenergic system and is involved in cognitive activity associated with emotional experiences. The relationship between synaptic Zn^2^^+^ signaling and the β-adrenergic system is an issue which requires further clarification. Stress is a known precipitant for metabolic and neurological diseases (Koenig et al., 2011) and synaptic Zn^2^^+^ signaling is likely to be involved in the diverse effects of stress through the stress mediators activated by CRH.

## PERSPECTIVE

Synaptic Zn^2^^+^ homeostasis is critical for synaptic function, and seems to be controlled by two Zn^2^^+^ pools, one in the synaptic vesicle and the other in the extracellular compartment. Synaptic Zn^2^^+^ signaling is involved in cognitive activity, and both its lack and its excess are involved in cognitive decline (**Figure 1**). HPA axis activity increases with aging, and this increase is superimposed on neurological diseases such as depression and Alzheimer’s disease. It is likely that synaptic Zn^2^^+^ signaling through the HPA axis activity is involved in cognitive decline in both normal aging and dementia, and it is possible that sympathetic nervous system activity is also involved. However, evidence related to synaptic Zn^2^^+^ dynamics is very limited, not only under physiological conditions, but also under stressful and pathological conditions. The molecular mechanisms of abnormal Zn^2^^+^ signaling in cognitive decline also remain to be clarified.

## Conflict of Interest Statement

The authors declare that the research was conducted in the absence of any commercial or financial relationships that could be construed as a potential conflict of interest.
